# Anorectal melanoma treated with abdominoperineal resection

**DOI:** 10.1002/ccr3.1784

**Published:** 2018-10-02

**Authors:** Shane M. Svoboda, Bashir Attuwaybi

**Affiliations:** ^1^ Sisters of Charity Hospital Buffalo New York

**Keywords:** abdominoperineal resection, anal cancer, anorectal melanoma, palliative surgery

## Abstract

The diagnosis of primary malignant melanoma of the anorectum is extremely rare and carries a very poor prognosis. While it is the third most common site of a primary melanoma, it accounts for <1% of anorectal malignancies. We present cases of anorectal melanoma treated with abdominoperineal resection.

## 1 INTRODUCTION

The diagnosis of primary malignant melanoma of the anorectum is extremely rare and carries a very poor prognosis. While it is the third most common site of a primary melanoma, it accounts for <1% of anorectal malignancies.^1^, ^2^

Diagnosis is often quite challenging due to its vague presentation. Rectal bleeding is the most common chief complaint. Unfortunately, this is often attributed to hemorrhoids, which delays the diagnosis. Patients may also complain of tenesmus or change in bowel habits. The importance of a high level of suspicion and a low threshold for biopsy of concerning lesions cannot be stressed enough.^3^

Prognosis in these cases remains extremely poor. Twenty percent of patients will have inguinal lymphadenopathy at presentation, while up to 25%‐35% will have distant metastasis.^1^, ^2^, ^4^ The five‐year survival rate has been reported to be approximately 6%; median survival is 12‐18 months.^4^, ^5^

The management of anorectal melanoma (ARM) has traditionally consisted of abdominoperineal resection (APR). However, evidence proving the survival benefit of such a morbid procedure is lacking; therefore, there has been a trend toward less invasive procedures such as wide local excision. Radiation and systemic chemotherapy have been utilized as a treatment of different variations. Results of systemic therapy, such as interferon, immunotherapy, and systemic chemotherapy, have been underwhelming.^8^

This article will review the presentation, management, and outcomes of two cases of primary anorectal melanoma at the same institution.

## 2 CASE REPORT A

Patient A is a 54‐year‐old female who initially presented to her primary care physician with the chief complaint of significant rectal pressure with a constant feeling of incomplete evacuation and rectal spasm. She also noted intermittent rectal bleeding, although this was attributed to hemorrhoids. She denied any changes in appetite or weight loss; family history was negative for colon cancer.

**Figure 1:**
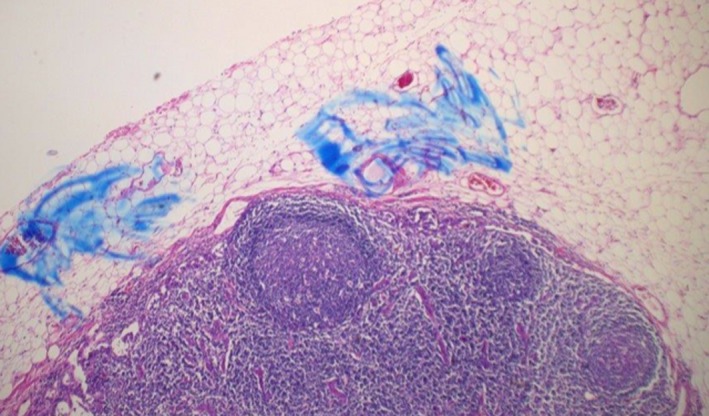
Case A histology

**Figure 2:**
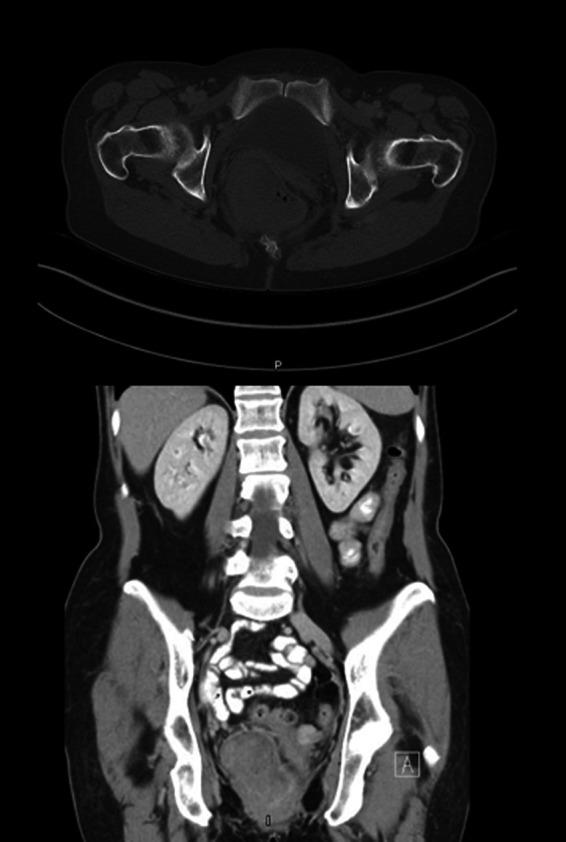
CT imaging case A

The patient was referred to the colorectal surgery service for a colonoscopy that revealed a large, malignant appearing rectal lesion. Biopsies were performed which confirmed melanoma, spindle cell type (Figure 1). She was then referred to a medical oncologist. The oncologic staging was performed, which consisted of a CT of the chest/abdomen/pelvis (Figure 2) and a bone scan. These studies were reviewed, and no definitive metastases were appreciated. The oncologist did not feel neoadjuvant therapy that was indicated and recommended surgical intervention. Given the size and involvement of the anal sphincters, an abdominoperineal resection (APR) was recommended.

**Figure 3:**
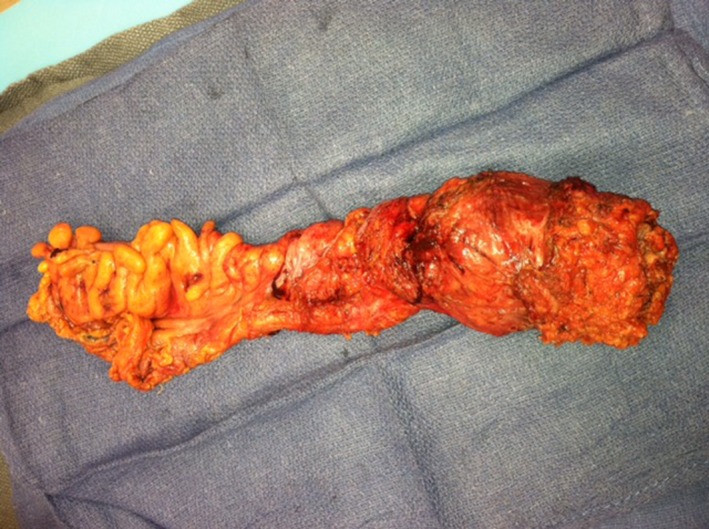
Case A surgical specimen

**Figure 4:**
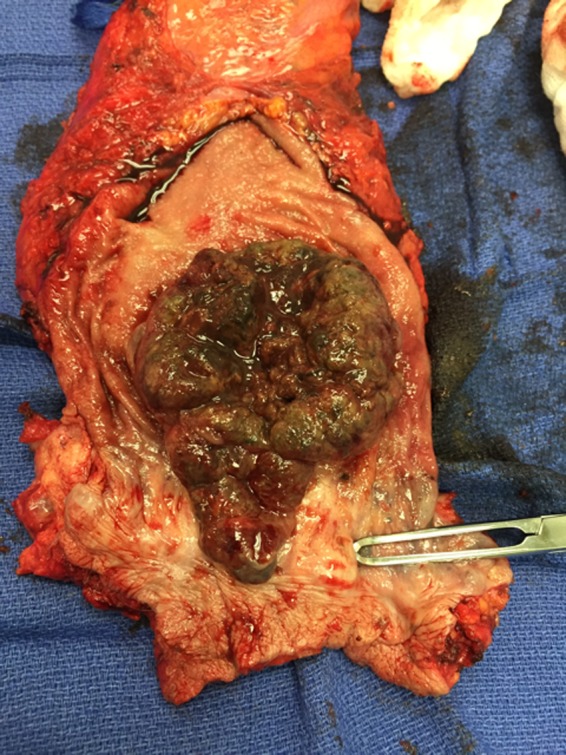
Case A gross pathology

The open abdominoperineal resection was performed without complication. Intra‐operatively, the involvement of the posterior vaginal wall was noted, and therefore, the patient underwent a posterior vaginectomy as well. Her postoperative course was uneventful. The final pathology revealed a 7 × 6 × 6 cm mass confined to the muscular wall consistent with high‐grade melanoma, 2/18 lymph nodes positive, pT2bN1bMx, negative margins, and no lymphovascular or perineural involvement (Figures 3 and 4). Immunohistochemistry with staining of S100, Melan A, and HMB‐45 was performed and positive for melanoma.

Approximately 1 month into her postoperative course, the patient sought a second opinion. A PET scan and CT of the brain were ordered. Unfortunately, metastatic involvement of the breast, liver, and lung was discovered. It was decided that systemic therapy was needed and high‐dose IL‐2 was recommended. After cycle 1 of high‐dose IL‐2 (600 000 IU/kg IV every 8 hours on days 1‐5 and 15‐19), restaging scan reveals the progression of the disease. This was confirmed with the findings of a new palpable posterior vaginal mass on pelvic examination at her 3‐month follow‐up appointment. The patient survived 4 months after initial diagnosis.

## 3 CASE REPORT B

**Figure 5:**
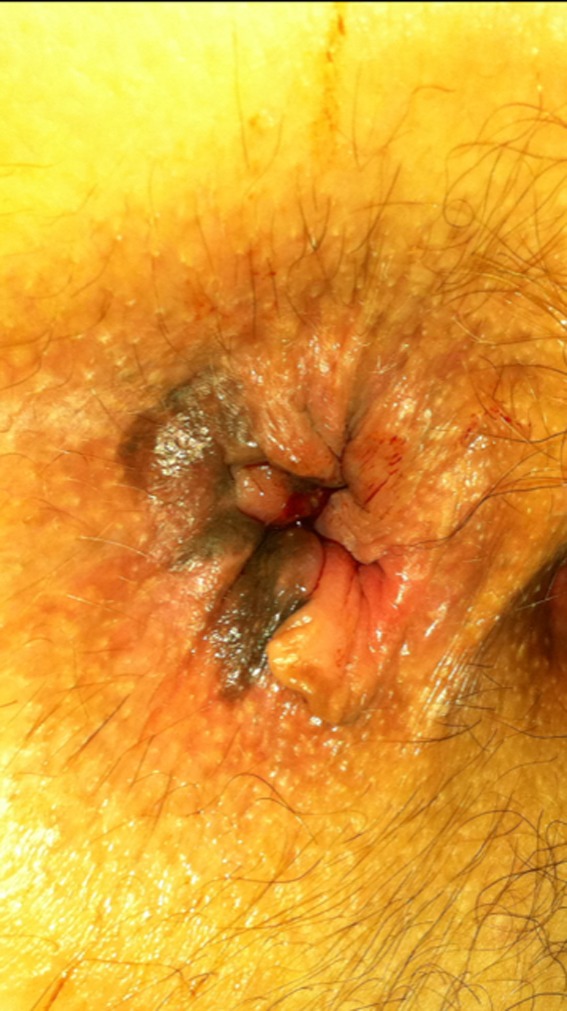
Case B external examination

**Figure 6:**
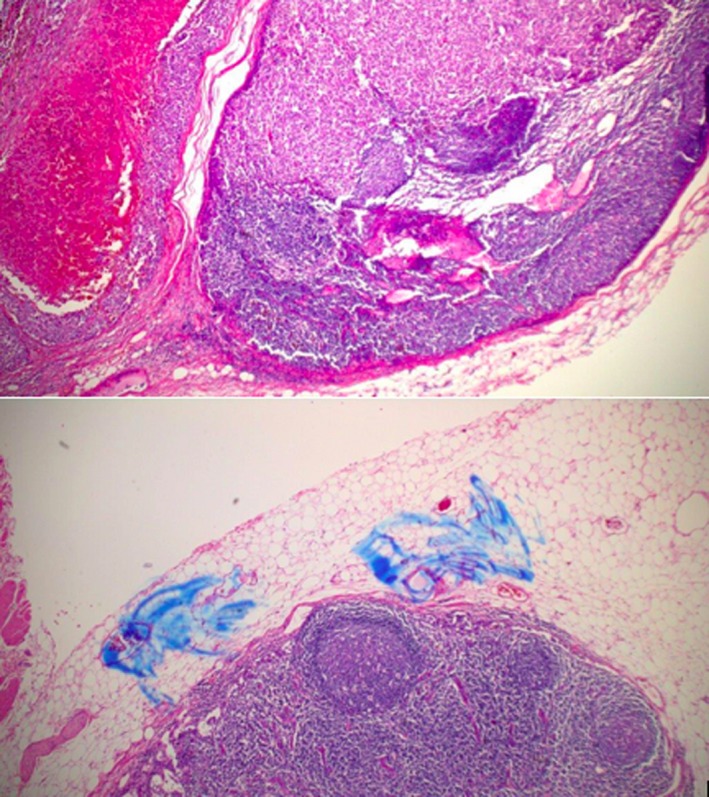
Case B histology

Patient B is an 84‐year‐old male referred for evaluation of constipation and rectal bleeding. Digital rectal examination revealed a posterior rectal mass (Figure 5). Colonoscopy, the following day, demonstrated a low‐lying ulcerated rectal mass. The final pathology report confirmed malignant melanoma (Figure 6).

An oncology referral was obtained at this point. CT of the chest, abdomen, and pelvis was performed at an outpatient radiology facility, which showed a large pelvic mass, along with multiple pulmonary nodules concerning for metastasis. There were no neoadjuvant or adjuvant therapies recommended. Surgical intervention was recommended as a modality of palliative therapy due to his extensive anorectal symptoms.

**Figure 7:**
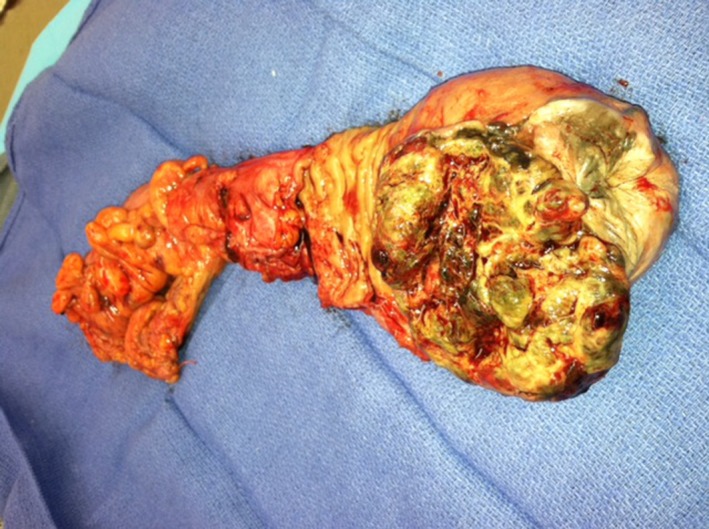
Case B surgical specimen

The patient underwent an open abdominoperineal resection. His immediate postoperative course was uneventful. The final pathology report revealed ulcerated malignant melanoma of the rectum measuring 8.7 x 7.2 x 3.2 cm, and tumor invades deep into muscularis propria, positive lymphatic and vascular invasion, 10/11 positive lymph nodes, and positive radial margins for final pathologic staging of pT3N1Mx (Figure 7). Immunohistochemistry staining with S100, Melan A, and HMB‐45 was performed and confirmed the diagnosis of melanoma.

Postoperative recovery progressed as expected, and he experienced significant symptomatic relief. The patient refused adjuvant chemotherapy after discussion of prognosis with medical oncology. A symptom‐based palliative approach was recommended by both medical oncology and colorectal surgery. The patient survived 5 months after the initial diagnosis.

## 4 DISCUSSION

As exhibited in our small case series, ARM carries a very poor prognosis. Definitive diagnosis is often delayed due to vague symptoms. The two cases above stress the importance of hypervigilance when evaluating a patient with rectal bleeding, regardless of their age. Anoscopy or flexible sigmoidoscopy should be utilized to rule out the most concerning etiologies, such as a melanoma in this case. Up to 35% of patients may present with metastatic disease; early detection may decrease this statistic.

Once the diagnosis of the ARM is pathologically confirmed and appropriate staging completed, surgery remains the mainstay of treatment.^4^, ^6^ Traditionally, APR was thought to be the appropriate approach for resection, regardless of the size and location of the lesion. Recent studies have shown similar survival rates between patients undergoing a wide local excision and those undergoing an APR. In both cases presented, the tumor was found to be large and bulky, therefore not amenable to local excision. An APR is the procedure of choice when the sphincters are involved; otherwise, the morbidity associated with an APR may outweigh the benefits. In patients without sphincter involvement, most clinicians would favor wide local excision (WLE). While negative margins are needed for an attempt at a curative resection, there are no clear guidelines established at this time in regard to surgical margins. The general trend seems to be 1 cm for a tumor with a depth <1 mm and 2 cm for deeper lesions.

Uncertainty remains in terms of the benefit of systemic adjuvant therapy and radiation therapy in the ARM. The use of radiation therapy in different modalities has been described, although no one regimen has proven superior.^7^ The use of systemic therapy has been underwhelming, as well. Dunn et al describe initial optimism for interferon a‐2b, although long‐term results eventually failed to show any survival benefit. The article goes on to stress that further studies are needed to determine the role of adjuvant treatments such as interferon, dacarbazine, and vaccine therapy.^4^

The two cases presented here demonstrate the severity of anorectal melanoma and share certain similarities that are not unique among this patient population. Presenting symptoms of each consisted of vague rectal pressure, along with rectal bleeding. While patient B sought medical attention quickly, patient A attributed the rectal bleeding to hemorrhoids. Despite initial uncertainty in the first case, patients A and B both presented with metastatic disease. Due to their severe symptoms of rectal bleeding and pain, an APR was required for palliation. Given the location of each lesion, an APR was the only option. However, if the location was more favorable, WLE could have been pursued. Immunomodulation, IL‐2, was utilized in our first patient, although the progression of disease was noted after the first cycle, which reiterates the fact that the role of systemic therapy has yet to be determined.

## 5 CONCLUSION

Anorectal melanoma is a rarely encountered malignancy that carries an extremely poor prognosis. Timely diagnosis is of paramount importance to decrease the chances of metastatic disease. Surgical intervention is the treatment of choice. The goal of therapy is to achieve negative margins. Due to the morbidity of an APR, WLE is favored when technically feasible. The benefit of radiation and systemic therapy remains unclear.

## CONFLICT OF INTEREST

None declared.

## AUTHORSHIP

SS: involved in the literature search and editing of manuscript, and preparation of images. BA: performed the initial manuscript author and provided relevant patient information.
